# SARS‐CoV‐2, bacterial co‐infections, and AMR: the deadly trio in COVID‐19?

**DOI:** 10.15252/emmm.202012560

**Published:** 2020-06-15

**Authors:** Jose A Bengoechea, Connor GG Bamford

**Affiliations:** ^1^ Wellcome‐Wolfson Institute for Experimental Medicine School of Medicine Dentistry and Biomedical Sciences Queen's University Belfast Belfast UK

**Keywords:** Microbiology, Virology & Host Pathogen Interaction

## Abstract

Respiratory viral infections are well known to predispose patients to bacterial co‐infections and superinfections. Still, there is limited reference to these in COVID‐19. Do co‐infections play a significant role during COVID‐19? What is the impact of antimicrobial resistance?

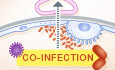

At the end of December 2019, Chinese public health authorities reported a cluster of pneumonia of unknown cause in central city of Wuhan in Hubei province. Shortly after, Chinese scientists identified a hitherto undescribed beta‐coronavirus as the likely causative agent. The disease is now referred to as coronavirus disease 2019 (COVID‐19), and the virus is called severe acute respiratory syndrome coronavirus 2 (SARS‐CoV‐2). Since 31 December 2019 and as of 25 May 2020, 5,432,512 cases of COVID‐19 (in accordance with the applied case definitions and testing strategies in the affected countries) have been reported globally, including 345,467 deaths. These numbers are very likely a significant under‐estimate of the true global burden of COVID‐19. To date, there are no licensed therapies nor vaccines to treat or protect against SARS‐CoV‐2 infection. Like the 1918 H1N1 influenza pandemic before it, COVID‐19 exemplifies the devastating impact of an emerging zoonotic pathogen on a susceptible “naïve” population.

In a significant number of infected individuals, SARS‐CoV‐2 infection leads to a mild upper respiratory tract disease or even asymptomatic “sub‐clinical” infection. However, published estimates indicate that the hospitalisation rate is higher than 8%, although it can be as high as 20% in the most at risk group (median age 72 years (preprint: Docherty *et al*, 2020)). A significant proportion of hospitalised patients require admission to intensive care units (ICU) with mortality rates reaching up to 50% for those ventilated patients. A picture is emerging in which male sex, increasing age and/or comorbidities such as diabetes, pulmonary disease or cardiovascular disease are associated with higher probability of mortality in severe COVID‐19 (preprint: Docherty *et al*, 2020), although it should be noted that roughly 50% of the hospitalised patients have no reported comorbidity.

Somewhat surprising is the limited reference to non‐viral co‐infections and super‐infections in COVID‐19. This knowledge gap is puzzling considering the extensive clinical evidence, supported by mechanistic studies in animal models, demonstrating that respiratory viral infections predispose patients to bacterial co‐infections and super‐infections. Thus, most fatalities in the 1918 influenza pandemic were indeed due to subsequent bacterial infection and similar observations were made during the later three 20th‐century influenza pandemics: the 1957 H2N2, the 1968–1969 H3N2 and the 2009–2010 H1N1 pandemics (Morris *et al*, 2017).

## Is there a case to consider co‐infections in COVID‐19?

We strongly believe co‐infections play a significant role during COVID‐19. Firstly, chronic obstructive pulmonary disease (COPD) is one of comorbidities associated with severe COVID‐19 (preprint: Docherty *et al*, 2020). COPD patients are colonised by bacterial pathogens even at the stable phase of the disease, making it likely that SARS‐CoV‐2 infection occurs in patients already colonised with bacteria. Secondly, the very likely possibility exists that severe COVID‐19 patients could be subsequently or co‐incidentally infected by bacteria. The median hospital stage of COVID‐19 patients is 7 days but can reach up to 14 days or even longer (preprint: Docherty *et al*, 2020), and the risk of a hospital‐associated pneumonia increases significantly the longer the hospitalisation period. Moreover, more than 90% of hospital‐associated pneumonias are associated with mechanical ventilation, being this intervention one of the therapeutics used in COVID‐19 patients admitted in the ICU. Not surprisingly, bacterial co‐infections were reported in MERS‐CoV patients receiving intensive care (Memish *et al*, 2020). Further supporting the need to consider co‐infections in COVID‐19, initial reports showed that 50% of the patients who have died had secondary bacterial infections (Zhou *et al*, 2020), and bacterial and fungal infections have been documented in severe COVID‐19 patients (Chen *et al*, 2020). However, the recent clinical study assessing features of COVID‐19 in more than 16,000 hospitalised patients in the United Kingdom (preprint: Docherty *et al*, 2020) does not include any reference to secondary infections despite being an item included in the ISARIC World Health Organization questionnaire used by the investigators. This may reflect the fact that co‐infections are largely not considered relevant during any large infectious epidemic in which the focus is set in the sole pathogen driving the outbreak and the identification of comorbidities to identify groups of patients at risk.

## What is the impact of co‐infections during COVID‐19?

**Figure 1:**
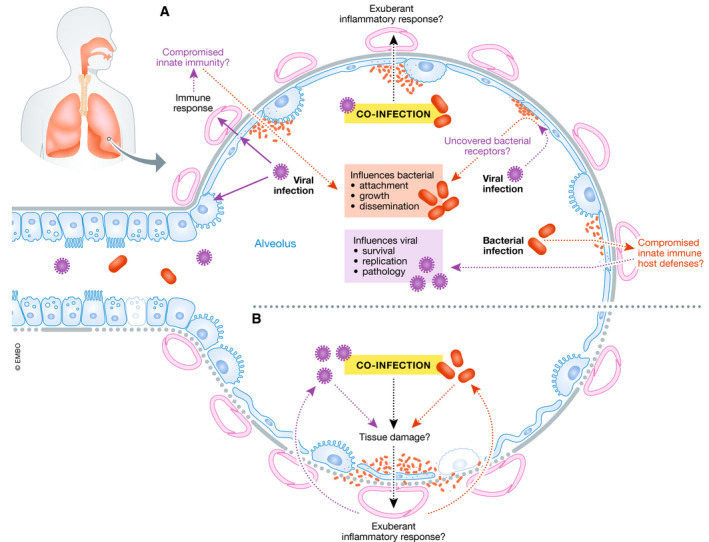
The interplay between SARS‐CoV‐2, bacteria and the host in co‐infections (A) SARS‐CoV‐2 virulence factors interact with the lungs and evoke an immune response. These interactions may compromise innate immunity at several levels resulting in increased bacterial attachment, growth and dissemination. Viral infection may uncover bacterial receptors mediating bacterial attachment. Co‐infection may result in an exuberant inflammatory response. It is also plausible that the type of immune response induced by SARS‐CoV‐2 may enable bacteria to flourish in the lungs. On the other hand, bacterial colonisation may predispose to SARS‐CoV‐2 infection because the innate immune host defences may be down‐regulated enabling virus survival, growth and pathology. (B) Co‐infection may exacerbate the tissue damage; and the exuberant inflammatory response may further amplify the lung damage triggered by SARS‐CoV‐2.

We envision three non‐mutually exclusive bacterial/SARS‐CoV‐2 co‐infections scenarios: 1) secondary SARS‐CoV‐2 following bacterial infection/colonisation; 2) combined viral/bacterial pneumonia; and 3) secondary bacterial “super‐infection” after SARS‐CoV‐2 (Fig 1). The underlying mechanisms of these scenarios are highly context and time‐dependent, involving complex interactions between three distinct entities (virus, host and bacteria). While it is clear that the immune response to SARS‐CoV‐2 is very likely not the same in a combined viral/bacterial pneumonia, altogether we postulate that ultimately any co‐infection scenario will worsen the clinical outcome and the severity of COVID‐19.

SARS‐CoV‐2 may enhance colonisation and attachment of bacteria to host tissue, and the combined infections may result in increased tissue destruction and pathophysiology. Airway dysfunction, cytopathology and tissue destruction induced by SARS‐CoV‐2 infection or during bacterial co‐infection may facilitate systemic dissemination of the virus and/or bacterial co‐pathogens, dramatically increasing the risk of blood infections and sepsis. Virus‐mediated enhancement of bacterial infection is not unprecedented. Rhinovirus and influenza virus infections increase the invasion of the airway epithelium by respiratory pathogens (Bosch *et al*, 2013).

Although the emerging evidence is suggestive of dysfunctional local and systemic antiviral and inflammatory responses during SARS‐CoV‐2 infection, the limited understanding of SARS‐CoV‐2 pathogenesis and its effect on immune signalling precludes us from speculating further (Tay *et al*, 2020). However, research on the very closely related SARS‐CoV has established that multiple viral structural and non‐structural proteins antagonise IFN responses (Versteeg *et al*, 2007). Reduced levels of type I IFNs are associated with increased susceptibility to secondary bacterial infections (Rynda‐Apple *et al*, 2015). It is expected that SARS‐CoV‐2 also deploys many proteins, such as the conserved NSP1, ORF6 and N, to blunt IFN production and signalling.

Bacterial co‐infections may even result in dampening of the activation of host defence signalling which may result in increasing susceptibility to SARS‐CoV‐2 infection and subsequent pathology. For example, respiratory pathogens including *Klebsiella pneumoniae* limit the activation of NF‐κB‐governed responses, which are also part of the host antiviral programme (Bengoechea & Sa Pessoa, 2019). Additionally, type I and III IFNs produced following bacterial infection may facilitate SARS‐CoV‐2 infection because the ACE2 receptor used by the virus is an IFN‐stimulated gene (Hoffmann *et al*, 2020), although whether IFN‐mediated ACE2 up‐regulation results in enhanced virus entry and infection is still unknown.

Another angle to consider is whether SARS‐CoV‐2 infection may perturb gut homeostasis, favouring bacterial respiratory infection. It is well established the important role of the gut–lung axis to control bacterial pneumonia (Dumas *et al*, 2018). Intriguingly, gastrointestinal symptoms are relatively common in COVID‐19 patients (preprint: Docherty *et al*, 2020), and SARS‐CoV‐2 has been shown to infect gut enterocytes *in vitro* and elicit an immune response (Lamers *et al*, 2020). Therefore, we suggest that it is very likely that the gut microbiota is perturbed in severe COVID‐19 patients which at the very least will affect disease outcomes including predisposition to secondary bacterial infections of the lung.

## COVID‐19 therapies and bacterial co‐infections

The lack of efficacious licensed therapies to treat severe COVID‐19 patients by targeting SARS‐CoV‐2 has led clinical colleagues to consider and trial drugs based on modulating the immune response, such as anti‐inflammatories to reduce the inflammation, and the use of biologics targeting some of the cytokines reported to be up‐regulated in patients such as IL1β, IL‐6 and TNFα (Tay *et al*, 2020). By no means do we question these early decisions but want to raise a cautionary tale because these immunomodulatory interventions may also increase the risk of potentially fatal secondary bacterial respiratory infections. For example, there is a significant increase of bacterial pneumonia in COPD patients treated with glucocorticoids (Calverley *et al*, 2007). Clinical evidence indicates that high doses of glucocorticoids increase the risk of secondary bacterial infections in patients with acute respiratory distress syndrome (ARDS) (Bernard *et al*, 1987). Dysregulated production and signalling of cytokines of the IL1 family, such as those found in COVID‐19 patients (Tay *et al*, 2020), can aggravate tissue damage during infection, making them potential attractive targets to treat severe COVID‐19. However, it should be noted that these cytokines and derived signalling pathways play a crucial role in host defence against bacterial respiratory pathogens. Perhaps not surprisingly, clinical studies have reported that although IL1 blockers are not associated with an increased risk of secondary infections, fatal infections occur more frequently during their treatment (Buckley & Abbate, 2018 *)*. Collectively, this evidence highlights the need to consider the impact of any intervention targeting inflammatory responses on secondary infections, necessitating through analysis on timing and dosing of administration of these drugs. Additional careful considerations should also be taken for recombinant cytokine therapy, such as treatment with type I or III IFNs, which could promote bacterial super‐infection and associated pathology.

## COVID‐19 and AMR

One dimension we feel has not received the necessary attention is the impact of COVID‐19 on antimicrobial resistance (AMR). Globally, the SARS‐CoV‐2 pandemic is superimposed on the ongoing pandemic of multidrug‐resistant bacteria. In the United Kingdom, according to the National Institute for Health and Care Excellence (NICE) guidance to treat severe pneumonia—irrespective of cause—patients will receive the broad‐spectrum antibiotics doxycycline or amoxicillin as an alternative. In most European countries, 15–50% of bacterial isolates are resistant to at least one antimicrobial group, and combined resistance to several antimicrobial groups is frequent, making it likely that the empirical broad antibiotic treatment will have limited effect on hospital‐acquired infections. In this scenario, nearly all severe COVID‐19 patients are treated with antibiotics, which may have limited efficacy. Worryingly, there is also clinical evidence suggesting that this inadequate broad‐spectrum empiric antibiotic use could be associated with higher mortality, at least in the case of sepsis (Rhee *et al*, 2020).

Unfortunately, as the pandemic continues, we anticipate a significant increase in AMR through the heavy use of antibiotics in COVID‐19 patients. Even in a normal scenario, ICUs are epicentres for AMR development. This may have devastating consequences in those hospital settings with already a high prevalence of multidrug‐resistant strains. It is evident that as SARS‐CoV‐2 is transmitting in hospitals, also multidrug‐resistant bacteria are, leading to an increase in the mortality due to the limited arsenal of antibiotics to treat hospital‐acquired infections. The treatment of patients after surgery, transplant or chemotherapy could be significantly compromised. In addition to the direct impact in the healthcare setting, the transmission of AMR to the environment should not be forgotten. For example, the increased levels of antimicrobials released in wastewater from hospitals will affect levels of antimicrobials in the environment, affecting the level of resistance in both animals (both wildlife and feed animals) and in farming and natural systems. Altogether, we believe that antibiotic stewardship principles should not be relaxed even in these challenging times. The need for antibiotic treatment should be rapidly evaluated and stopped if not necessary. We do not advocate its prophylactic use. Ideally, if antibiotics are needed, the microbiology laboratory should inform which ones are the most suitable based on the microorganism and the resistance pattern.

One final aspect to consider is the enhanced use of hand sanitisers and antibacterial soaps as measures of protection against SARS‐CoV‐2. While we do not argue against this practice, we stress that it is important to note that some of them may content additional chemicals that do not add much in terms of protection but instead may fuel bacterial antimicrobial resistance. Bacteria exploit efflux pumps to develop resistance against disinfectants, and these same efflux pumps contribute to AMR. In any case, it is essential that the public adhere to the manufacturer's instructions for proper use to avoid the selection of bacteria with increased tolerance/resistance to antimicrobials.

## Future directions

In this quickly evolving field, there is still an urgent need to identify and characterise bacterial co‐infections during COVID‐19. ARDS is one of the well‐established features of COVID‐19 pathophysiology, and it is recognised the increasing risk of nosocomial pneumonia during the course of ARDS. Moreover, pathological analysis of post‐mortem biopsies of lung from patients who died of severe COVID‐19 revealed histopathologic findings consistent with superimposed bacterial pneumonia in some patients (Tian *et al*, 2020). Early and rapid diagnosis using metagenomic next‐generation sequencing‐based approaches will allow the detection of a broad range of pathogens (including their antimicrobial resistance profiles) and will undoubtedly contribute to necessary enhanced antibiotic stewardship. However, we also believe it is imperative to generate a molecular understanding of the causes and consequences of bacterial co‐infections during COVID‐19 in order to aid the development of novel therapeutic interventions influencing targets with high efficacy and safety during co‐infection. This investigation should exploit established relevant preclinical research models such as well‐differentiated airway epithelial and immune cell systems alongside characterised and pathogenic viral and bacterial isolates. In sum, it is critical that co‐infections should not be underestimated and instead be part of an integrated and holistic plan to limit the global burden of morbidity and mortality during the SARS‐CoV‐2 pandemic and beyond. We hope that exploring the role of bacterial and SARS‐CoV‐2 co‐infections will result in both the improved health of COVID‐19 patients but also uncover exciting new biology of the three‐way “trans‐kingdom” interactions between viral and bacterial pathogens within the host respiratory mucosae.
